# Reverse-Transcriptase PCR Detection of *Leptospira*: Absence of Agreement with Single-Specimen Microscopic Agglutination Testing

**DOI:** 10.1371/journal.pone.0132988

**Published:** 2015-07-15

**Authors:** Jesse J. Waggoner, Ilana Balassiano, Alisha Mohamed-Hadley, Juliana Magalhães Vital-Brazil, Malaya K. Sahoo, Benjamin A. Pinsky

**Affiliations:** 1 Department of Medicine, Division of Infectious Diseases and Geographic Medicine, Stanford University School of Medicine, Stanford, California, United States of America; 2 Laboratório de Zoonoses Bacterianas, Centro de Referência Nacional para Leptospirose, Coleção de Leptospira, WHO/PAHO Centro Colaborador para Leptospirose, Instituto Oswaldo Cruz, Fiocruz, Rio de Janeiro, Brazil; 3 Department of Pathology, Stanford University School of Medicine, Stanford, California, United States of America; University of Kentucky College of Medicine, UNITED STATES

## Abstract

**Background:**

Reference diagnostic tests for leptospirosis include nucleic acid amplification tests, bacterial culture, and microscopic agglutination testing (MAT) of acute and convalescent serum. However, clinical laboratories often do not receive paired specimens. In the current study, we tested serum samples using a highly sensitive real-time nucleic acid amplification test for *Leptospira* and compared results to MAT performed on the same specimens.

**Methods/Principal Findings:**

478 serum samples from suspected leptospirosis cases in Rio de Janeiro were tested using a real-time RT-PCR for the diagnosis of leptospirosis, malaria and dengue (the Lepto-MD assay). The Lepto-MD assay detects all species of *Leptospira* (saprophytic, intermediate, and pathogenic), and in the current study, we demonstrate that this assay amplifies both *Leptospira* RNA and DNA. Dengue virus RNA was identified in 10 patients, and no cases of malaria were detected. A total of 65 samples (13.6%) were positive for *Leptospira*: 35 samples (7.3%) in the Lepto-MD assay, 33 samples (6.9%) by MAT, and 3 samples tested positive by both (kappa statistic 0.02). Poor agreement between methods was consistent regardless of the titer used to define positive MAT results or the day of disease at sample collection. *Leptospira* nucleic acids were detected in the Lepto-MD assay as late as day 22, and cycle threshold values did not differ based on the day of disease. When Lepto-MD assay results were added to the MAT results for all patients in 2008 (n=818), the number of detected leptospirosis cases increased by 30.4%, from 102 (12.5%) to 133 (16.3%).

**Conclusions/Significance:**

This study demonstrates a lack of agreement between nucleic acid detection of *Leptospira* and single-specimen MAT, which may result from the clearance of bacteremia coinciding with the appearance of agglutinating antibodies. A combined testing strategy for acute leptospirosis, including molecular and serologic testing, appears necessary to maximize case detection.

## Introduction

Leptospirosis is a potentially fatal zoonotic disease that occurs throughout the world, but reported incidence rates likely underestimate the true burden of disease [1,2]. This situation results from many factors, but it is due, in part, to limitations of available diagnostics to accurately detect leptospirosis in the acute setting [3]. Laboratory confirmation of leptospirosis requires one of the following: a four-fold increase in antibody titer between acute and convalescent serum samples, as detected by microscopic agglutination testing (MAT); a high MAT titer (≥ 1:400 to 1:800) in single or paired serum samples; isolation of pathogenic *Leptospira* species from a normally sterile site; or the detection of DNA from pathogenic *Leptospira* species by PCR [2,4]. Outside of a structured research setting, it can be difficult to obtain paired specimens for serologic testing [5]. Laboratories often receive only one sample from a suspected case, which leaves single-specimen MAT and molecular diagnostics as the principal means of confirming leptospirosis.

The Centro de Referência Nacional para Leptospirose (CRNL) at the Instituto Oswaldo Cruz, Fiocruz, in Rio de Janeiro receives 500 to 1,000 serum samples from patients in the Rio de Janeiro State annually for reference *Leptospira* testing. In the majority of cases (~90%), only a single specimen is received for testing by MAT, conventional PCR, and/or bacterial culture. Recently, our group reported the development of two molecular diagnostics for *Leptospira*: 1) an internally-controlled, multiplex assay for detection of all species of *Leptospira*, *Plasmodium* species with a specific call-out for *P*. *falciparum*, and the four dengue virus (DENV) serotypes (referred to here as the Lepto-MD assay, previously described as the UFI assay) [6]; and 2) an assay for the detection of pathogenic *Leptospira* species [7]. These assays target a region of the 16S *rrs* gene, and using a set of 65 serum samples from CRNL, both proved more sensitive than a reference *Leptospira* 16S PCR [7]. Of these samples, 55 had been tested by MAT, including only 6 positives, which limited our ability to compare test results from these two methods. Also, given the design of the Lepto-MD and pathogenic *Leptospira* assays, it was possible that improved sensitivity resulted from RNA amplification with or without concomitant DNA amplification.

In the current study, we tested a larger set of serum samples, collected in Rio de Janeiro in 2008, to compare results of screening with the Lepto-MD assay and single-specimen MAT for the detection of leptospirosis cases. The improved clinical sensitivity of the Lepto-MD assay, compared to other molecular tests, was utilized to identify missed cases of leptospirosis in this population and generate a new estimate of leptospirosis incidence for the study period. Finally, we evaluated the contribution of *Leptospira* RNA detection to the performance of the Lepto-MD and pathogenic *Leptospira* assays.

## Methods

### Ethics Statement

The study protocol was reviewed and approved by the Stanford Institutional Review Board and the Scientific Review Board of Instituto Oswaldo Cruz, Fiocruz, Rio de Janeiro, Brazil (CAAE:32200914.2.00005248).

### Clinical Samples and MAT

Archived, de-identified serum samples were selected for RT-PCR from specimens that had been sent from patients in Rio de Janeiro to CRNL for reference *Leptospira* testing between January 1 and December 31, 2008. During this period, CRNL received 894 serum samples for MAT from 818 patients in Rio de Janeiro: 749 patients had one sample, 62 patients had two samples, and 7 patients had three samples sent for testing. Samples were obtained as part of routine care from suspected leptospirosis cases. Day of disease at sample collection, when provided, was recorded by the requesting provider. All samples were tested upon receipt at CRNL by MAT using a reference panel of 19 *Leptospira* serovars, as described [7,8]. A positive MAT result was defined as any one of the following: a negative acute-phase titer and a follow-up sample with a titer ≥1:100; a 4-fold rise in titer by MAT between paired samples; or a single acute-phase MAT titer of ≥1:800. For analyses involving a comparison of test results for *individual samples*, samples were considered positive by MAT if the agglutination titer was ≥ 1:800 and indeterminate if the titers were 1:100–1:400. Up to 55 samples per month with at least 200μL of volume were selected for molecular testing. If sufficient numbers were available from a given month, only MAT-negative samples were selected. MAT-positive samples were included from months with <55 MAT-negative samples.

### Molecular Testing

Serum was stored at -20°C at CRNL until shipment to Stanford. Total nucleic acids were extracted from 200μL of serum using an easyMAG instrument (Biomerieux) with a 60μL elution volume. Two eluate aliquots were stored at -80°C. All samples were screened using the Lepto-MD assay, which was performed and interpreted as previously described [6]. Samples positive for *Leptospira* in the Lepto-MD assay were tested using three additional assays: 1) a 16S RT-PCR for the detection of pathogenic *Leptospira* species [7]; 2) the *Leptospira* 16S PCR, originally reported by Smythe, et al. [9]; and 3) a PCR for the detection of *lipL32* [10]. The latter two assays were performed as described elsewhere [11]. Results were analyzed on the linear scale, threshold was set at 0.05, and samples were considered positive in either assay if an exponential curve crossed the threshold prior to cycle 45. All molecular tests were performed on a Rotor-Gene Q instrument (Qiagen) using 5μL of eluate.

Results of MAT and molecular testing were compared for individual samples based on the timing of sample collection and cutoff used to define a positive MAT result. Samples obtained at ≤7 days of disease, 8–14 days, and >14 days were categorized as acute, late-acute, and convalescent, respectively. Results were organized by month of the year, and the proportion of positive tests was evaluated in relation to recorded rainfall. Data regarding precipitation in Rio de Janeiro during the study period was obtained from the Armazém de Dados, Portal da Prefeitura da Cidade do Rio de Janeiro (armazemdedados.rio.rj.gov.br, accessed 5 March 2015).

### PCR Evaluation and RNase A Treatment

The sensitivity of *Leptospira* DNA detection was evaluated using two real-time PCR kits: 1) the TaqMan Universal PCR Master Mix and 2) Platinum *Taq* DNA Polymerase (both from Life Technologies). The *Leptospira* and RNase P primers and probes from the Lepto-MD assay were used at the same final reaction concentrations with both real-time PCR kits. Real-time PCRs were evaluated by testing extracted nucleic acids from cultured isolates of *L*. *interrogans* serovar Australis (reference strain Ballico), *L*. *biflexa* serovars Patoc (reference strain Patoc I) and Andamana (reference strain CH 11), *L*. *borgpetersenii* serovar Castellonis (reference strain Castellon 3), *L*. *kirschneri* serovar Grippotyphosa (reference strain Moskva V), and *L*. *weilii* serovar Vughia (reference strain LT 89–68) [6]. Nucleic acids were extracted from cultured isolates using the DNeasy Blood & Tissue Kit (Qiagen) according to manufacturer recommendations. Real-time *Leptospira* PCR performance was compared to the Lepto-MD assay [6].

RNase A treatment was performed on extracted nucleic acids from the cultured *Leptospira* isolates, cultured strains of DENV-1 (Hawaii 1944) and DENV-2 (New Guinea C), plasmid DNA containing the *Leptospira* target sequence, and synthetic ssDNA containing the target sequence for DENV-1. To each 25uL of nucleic acid, RNase A (Thermo Scientific) was added to a final concentration of 100ug/mL. Samples were incubated at 37°C for 1hr and then placed on ice. Water was added to control aliquots of each sample. Treated samples and controls were immediately run side-by-side in the Lepto-MD assay.

### Statistics

A confirmed case of leptospirosis required nucleic acid detection by RT-PCR and/or a positive result by MAT. Basic statistics were calculated using Excel software (Microsoft). GraphPad software (GraphPad) was used for the calculation of kappa-statistics, two-tailed Fisher’s exact tests, and t-tests. Kappa-statistics were calculated to evaluate the agreement between MAT and the Lepto-MD assay for individual samples. Fisher’s exact tests were used in comparisons of categorical variables, and t-tests were performed for comparisons of continuous variables. Significance was defined as a p-value ≤ 0.05.

## Results

### Lepto-MD Assay Testing

A total of 479 serum samples from suspected leptospirosis cases were extracted and tested in the Lepto-MD assay. An assay for RNase P detection serves as the internal control in the Lepto-MD assay [6]. A single internal-control failure occurred in a sample from November; this was excluded from further analysis. Results are shown in Table 1 for the remaining 478 samples (99.8%) based on the month in which the specimen was obtained. No samples tested positive for *Plasmodium* species. DENV was detected in 10 samples (2.1%). Compared to leptospirosis cases (Table 2), patients with DENV were younger (p = 0.028), more likely to be female (p≤0.001), and presented earlier in the course of their illness [mean day of disease, 4.6 (standard deviation 2.4) versus 12.3 (standard deviation 7.9), respectively; p = 0.015). This latter association remained significant if leptospirosis cases that were only detected by MAT were excluded from the analysis (p = 0.028). No DENV and *Leptospira* co-infected patients were detected.

**Table 1:** Total number of samples tested from 2008 and positive results in the Lepto-MD assay by month.

			Positive Samples	
Study Month	Samples Tested	MAT Positive	Dengue virus	*Leptospira*
	n	n	n (%)	n (%)
January	55	0	1 (1.8)	3 (5.5)
February	23	2	2 (8.7)	2 (8.7)
March	55	0	5 (9.1)	5 (9.1)
April	50	4	0 (0)	2 (4.0)
May	18	3	0 (0)	2 (11.1)
June	55	0	2 (3.6)	4 (7.3)
July	54	0	0 (0)	6 (11.1)
August	36	1	0 (0)	2 (5.6)
September	8	1	0 (0)	1 (12.5)
October	49	8	0 (0)	4 (8.2)
November^1^	40	8	0 (0)	1 (2.5)
December	35	6	0 (0)	3 (8.6)
**Total**	478	33 (6.9)	10 (2.1)	35 (7.3)

^1^ Forty-one samples were extracted in November. One sample had a failed internal control reaction and was not included in further analyses.

**Table 2:** Patient characteristics associated with 478 samples that tested positive and negative for *Leptospira*.

	*Leptospira* Positive			*Leptospira* Negative	
	Total^1^	Lepto-MD Assay	MAT	Total	DENV-positive
Number, n	65	35	33	413	10
Male gender, n (%)	55 (84.6)	28 (80.0)	27 (90.0)	253 (61.3)	3 (30.0)
Age, years, mean (SD)	36.7 (15.3)	35.6 (17.0)	38.0 (13.4)	32.8 (18.4)	23.2 (20.2)
Day of Disease, n (%)					
Acute	12 (18.5)	8 (22.9)	4 (12.1)	115 (27.8)	6 (60.0)
Late Acute	11 (16.9)	6 (17.1)	5 (15.2)	58 (14.0)	1 (10.0)
Convalescent	13 (20.0)	4 (11.4)	10 (30.3)	73 (17.7)	0 (0.0)
Not provided	29 (44.6)	17 (48.6)	14 (42.4)	167 (40.4)	3 (3.0)

Abbreviations: SD, standard deviation

^1^ 65 samples tested positive for *Leptospira* in the Lepto-MD assay and/or by MAT; 3 samples tested positive by both methods.

### Leptospirosis Cases

A total of 65 samples (13.6%) were positive for *Leptospira*. *Leptospira*-positive patients were more likely to be male than patients who tested negative (p≤0.001), but the day of disease at sample collection did not differ significantly between these groups. Thirty-five samples (7.3%) tested positive in the Lepto-MD assay and 33 samples (6.9%) were positive by MAT (Table 2). Only three samples tested positive by both the Lepto-MD assay and MAT (Table 3A, kappa statistic 0.02), demonstrating poor agreement between these methods. MAT thresholds of ≥1:100 and ≥1:1600 did not affect concordance between Lepto-MD assay and MAT results (kappa 0.05 and -0.03, respectively; Table 3B and 3C). Information regarding the day of disease at sample collection was available for 282 samples. Samples that tested positive in the Lepto-MD assay were collected significantly earlier than samples positive by MAT [mean day of disease, 9.5 (standard deviation 5.3) vs. 15.0 (standard deviation, 8.8); p ≤ 0.03]. However, poor agreement between the Lepto-MD assay and MAT was consistent regardless of the day of disease at sample collection. Of the samples collected at ≤ 14 days of illness, 14 tested positive in the Lepto-MD assay, 9 were positive by MAT, but none had concordant results between these methods. A single sample collected on day 16 and two samples without recorded day of disease information tested positive by both methods.

**Table 3:** Comparison of *Leptospira* results for 478 serum samples tested with the Lepto-MD assay and MAT using titer thresholds for a positive result of ≥1:800 (definition of a confirmed case, ≥1:100, and ≥1:1,600.

		Lepto-MD Assay		
		Positive	Negative	Total
**MAT ≥1:800**	Positive	3	30	33
	Negative	32	413	445
**MAT ≥1:100**	Positive	5	38	43
	Negative	30	405	435
**MAT ≥1:1,600**	Positive	1	23	24
	Negative	34	420	454
	Total	35	443	478

**Fig 1:**
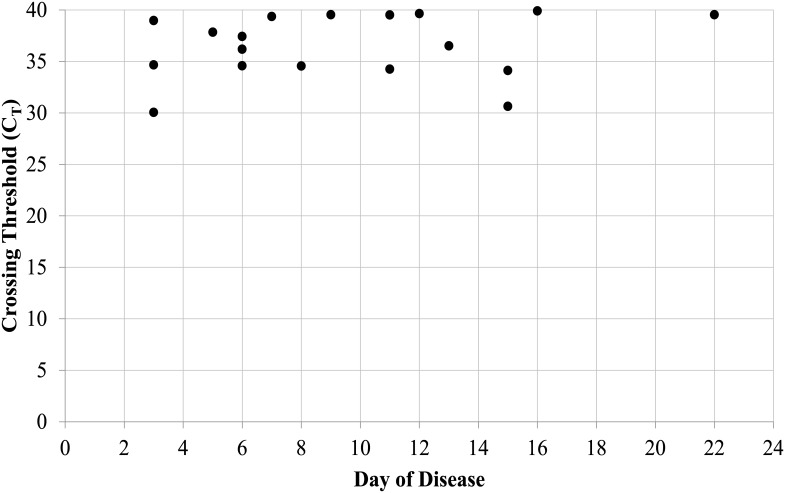
*Leptospira* C_T_ values in the Lepto-MD assay for patients with recorded day of disease information.

*Leptospira* was detected in the Lepto-MD assay from samples collected as late as day 22. Rates of detection in the Lepto-MD assay did not differ significantly for samples collected in the acute [8/127 (6.3%)], late acute [6/69 (8.7%)], or convalescent periods [4/86 (4.7%); p≥0.05 for each comparison, Fisher’s exact test]. Cycle threshold (C_T_) values in the Lepto-MD assay also did not differ based on the day of disease at sample collection (Fig 1), and mean C_T_ values were similar for samples with recorded day of disease information and those without this information [36.5 (standard deviation 3.1) vs 34.4 (standard deviation, 5.1); p ≤ 0.15). In order to confirm *Leptospira* results from the Lepto-MD assay, 33/35 (94.3%) positive samples were tested using three other molecular tests (see Methods). Two samples had insufficient volume for all comparator tests. 27 of these 33 samples (81.8%) tested positive for *Leptospira* in at least one other assay, including 25/27 (92.6%) that tested positive for pathogenic species.

**Fig 2:**
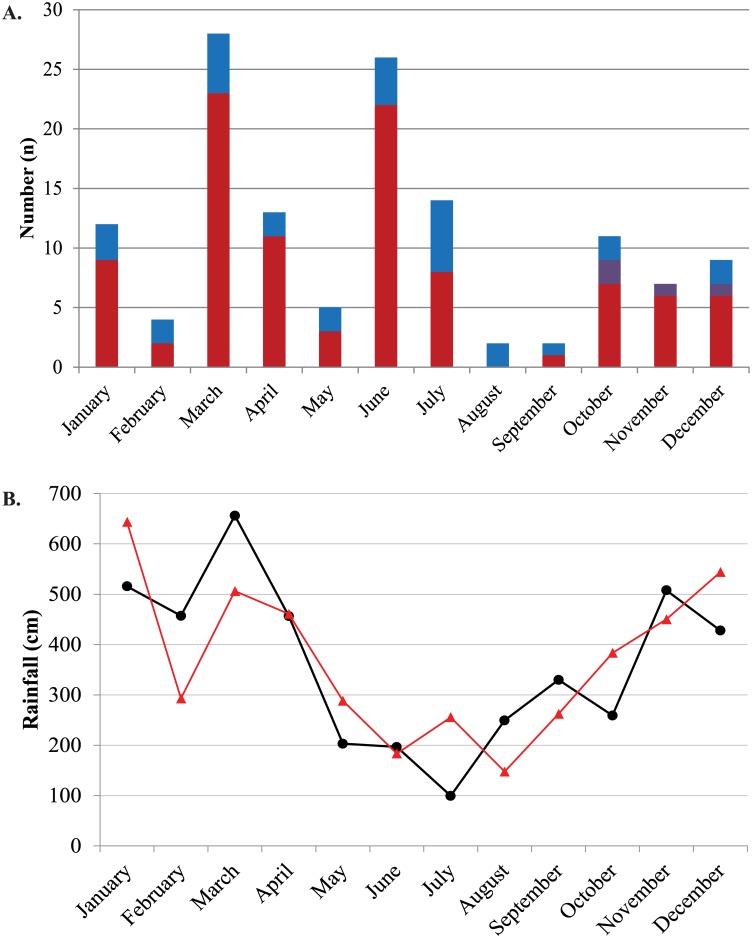
Leptospirosis cases and recorded rainfall in Rio de Janeiro by study month. (A) Leptospirosis cases diagnosed by RT-PCR (blue), MAT (maroon), or both (purple) are displayed by study month. MAT results are shown for all samples from 2008. (B) Rainfall for 2008 (black circles and line) and the average rainfall for the years 2003–2013 (red triangles and line).

Leptospirosis case detection using the Lepto-MD assay was then added to MAT results for all samples sent to CRNL from Rio de Janeiro during 2008 (Fig 2A). 818 patients had MAT performed, and 102 (12.5%) tested positive. In 77 cases (75.5%), antibody titers were highest to serovars Icterohaemorrhagiae and Copenhageni. From the patients who had samples tested for this study, one additional case was detected by seroconversion when all MAT results were analyzed. The addition of Lepto-MD test results, therefore, increased the number of detected leptospirosis cases by 30.4%, from 102 (12.5%) to 133 (16.3%). The highest number of cases was detected in March, which was also the month with the highest recorded rainfall (Fig 2B). However, there was not a clear seasonality to *Leptospira* detection using either RT-PCR or MAT despite normal rainfall patterns in Rio de Janeiro during the study period (Fig 2B).

### PCR versus RT-PCR

An internally-controlled *Leptospira* real-time PCR was evaluated using Platinum *Taq* DNA Polymerase (Life Technologies) and extracted nucleic acids from 6 control *Leptospira* strains. This PCR uses the same *Leptospira* primers and probes as the Lepto-MD assay at the same final reaction concentrations. Samples were tested side-by-side with the Lepto-MD assay, which is performed using the SuperScript III Platinum One-Step qRT-PCR kit (Life Technologies). C_T_ values in the PCR were, on average, 5.6 cycles later than C_T_ values in the Lepto-MD RT-PCR assay. Results were similar using the TaqMan Universal PCR Master Mix (also Life Technologies), and the use of three-step versus two-step cycling did not change these findings. To confirm that the improved performance of the Lepto-MD assay was due to the detection of RNA, nucleic acid aliquots from *Leptospira* controls were treated with RNase A. C_T_ values for treated samples were 10.8 cycles later than untreated controls. RNase A treatment had a larger effect on DENV RNA controls (C_T_ difference 22.7 cycles) and no demonstrable effect on DENV-1 ssDNA or *Leptospira* plasmid DNA (C_T_ difference 0.1 cycles).

## Discussion

This study describes the results of testing 478 serum samples from suspected leptospirosis cases in Rio de Janeiro with the Lepto-MD assay. Thirty-five cases of leptospirosis and 10 dengue cases were identified. It is interesting to note that dengue cases were significantly younger than cases of leptospirosis. This has not been observed during previous studies of concurrent DENV and *Leptospira* outbreaks [12–14]. The current study involves a relatively small number of cases, however, and this will need to be confirmed in a larger number of patients. No cases of malaria were identified in this population, which is consistent with the epidemiology of malaria in Brazil [15].

Molecular detection of *Leptospira* in the Lepto-MD assay demonstrated poor agreement with single-specimen MAT. We employed a conservative threshold for calling positive samples by MAT (titer ≥ 1:800), which is consistent with studies by other researchers in Brazil and was selected to maximize specificity [16–18]. Poor agreement between molecular testing and MAT remained consistent when different titer thresholds were evaluated and when results were stratified by the day of disease at sample collection. These findings could result from poor specificity of either the Lepto-MD assay or single-specimen MAT, though this seems unlikely. Of samples that tested positive for *Leptospira* in the Lepto-MD assay, 27/33 (81.8%) were confirmed with another molecular test. Although six samples were not confirmed by another molecular test, this is consistent with the improved sensitivity of the Lepto-MD assay relative to these comparators [7]. The Lepto-MD assay also demonstrated good specificity during the initial clinical evaluation and when used in patient populations with a low incidence of leptospirosis [6,19]. MAT is regarded as a highly specific test, though few studies report the clinical specificity of MAT performed on a single specimen. In a study by Cumberland, et al., MAT had a specificity of 99% compared to bacterial culture, even in the first, acute phase specimens [20]. This group also used an MAT titer of ≥1:800 to define a positive test. A more recent evaluation of *Leptospira* diagnostics employed latent class analysis to estimate the specificity of MAT at 98.8% [21]. A titer of ≥1:400 defined as a positive result, but specificity of single-specimen MAT was not evaluated as a separate end-point.

The classic description of leptospirosis is an acute systemic febrile illness with a biphasic course corresponding to an early leptospiremic phase and a later immune phase. The lack of agreement between molecular testing and single-specimen MAT likely resulted from the clearance of leptospiremia coinciding with the appearance of agglutinating antibodies in serum during the immune phase of illness [2,3,22]. This is consistent with the earlier collection of samples that tested positive in the Lepto-MD assay compared to MAT-positive samples. Interestingly, *Leptospira* detection in the Lepto-MD assay occurred as late as day 22, and samples collected as early as day 1 were positive by MAT. While inaccurate recording of the day of disease at sample collection could affect these numbers, the prolonged detection of *Leptospira* nucleic acids in serum has been observed by our group as well as others who utilized different molecular assays in varied patient populations [7,23,24]. Taken together, these findings argue that some patients may present acutely, though symptom onset occurred during the immune phase of disease, while others may fail to generate the expected antibody response and develop prolonged bacteremia. Given these two potential disease courses, there is a limit to the clinical sensitivity attainable with molecular diagnostics and a continued role for serologic testing in the workup of acute leptospirosis. Early diagnosis may also be aided by performing molecular testing on non-blood specimen types, such as urine and cerebrospinal fluid, where *Leptospira* can be detected later in the disease course [2,19].

While testing of acute and convalescent serum is recommended for the diagnosis of leptospirosis by MAT, such specimens can be difficult to obtain in a typical laboratory setting. Of the 818 patients who had serum sent to CRNL for testing in 2008, only 69 (8.4%) had multiple specimens collected. This is similar to findings from the Royal Tropical Institute in The Netherlands, where, in a study detailing testing results from 2001 to 2012, only 18.1% of patients had multiple samples available for serology [5]. However, a majority of patients with confirmed leptospirosis actually had paired specimens (86.9%), illustrating the need for such specimens to improve the sensitivity of MAT. In the absence of paired samples, though, molecular testing can significantly increase leptospirosis case detection rates. If all MAT-negative samples in our population had been tested with the Lepto-MD assay, for example, the predicted incidence of leptospirosis would increase 50%, from 102 cases to 153 cases (12.5% to 18.7%, respectively, p<0.001). It will be useful to evaluate such a combined testing strategy, as it may capture all or almost all of the cases that could be detected using paired samples by MAT [21].

Finally, this study demonstrates the increased sensitivity of *Leptospira* detection in the Lepto-MD assay, performed as a real-time RT-PCR, compared to an optimized real-time PCR using the same *Leptospira* primers and probes. The Lepto-MD assay targets the *Leptospira* 16S *rrs* gene, and based on our findings using control *Leptospira* isolates, detection of both RNA and DNA occurs. Further experiments directly comparing PCR and RT-PCR on *Leptospira* positive clinical specimens with and without RNase A treatment will be required for full characterization. However, the current study lacked sufficient remaining nucleic acids extracted from positive clinical specimens to perform this testing. Of note, nucleic acids from the control *Leptospira* isolates were extracted using the DNeasy Blood & Tissue Kit (Qiagen). While optimized for DNA extraction, this kit does not specifically remove RNA from the eluted nucleic acids. The increased sensitivity of RT-PCR for the detection of a bacterial 16S target may prove useful in the design of assays for other pathogens that are present at low levels in the blood. Importantly, such assays may not require the use of new or automated extraction protocols.

In conclusion, this study demonstrates a lack of agreement between molecular detection of *Leptospira* and single-specimen MAT, regardless of the titer cut-off or day of disease at sample collection. Positive results from these methods demonstrate virtually no overlap, and a combined testing strategy for acute leptospirosis, including a sensitive molecular diagnostic and serologic testing, appears necessary to maximize case detection. Furthermore, we have demonstrated improved sensitivity of *Leptospira* detection using real-time RT-PCR; similar test designs may prove useful for other systemic bacterial infections that are challenging to diagnose.
